# Supplementary material for: Splice-Junction-Based Mapping of Alternative Isoforms in the Human Proteome
Source: Cell Rep. Author manuscript; Available in PMC 2020 Jan 15. (PMC6961840; doi:10.1016/j.celrep.2019.11.026)
Supplement: 2 [file NIHMS1546469-supplement-2.zip › DF1/PXD010154/Testis/Testis_55_ATP6V1B2_ASAIQFLQFPLVNGAAPELPVPTGGPAVGAR.pdf]

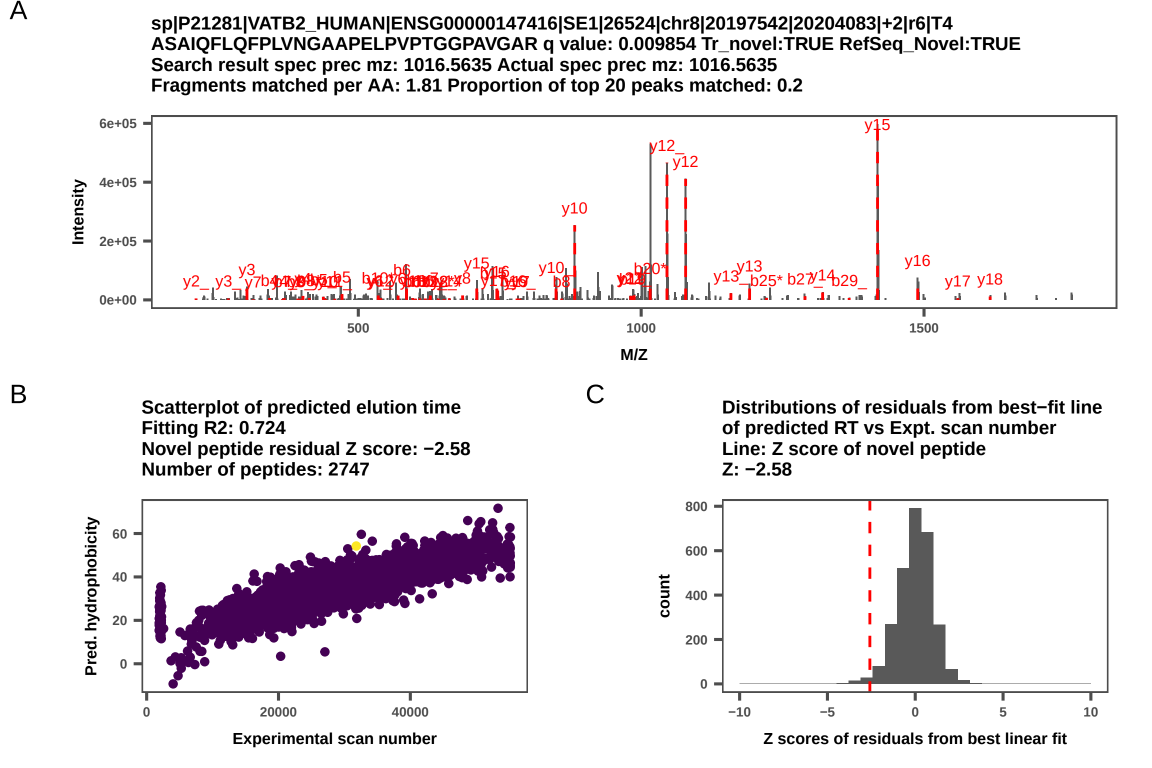
A
sp|P21281|VATB2_HUMAN|ENSG00000147416|SE1|26524|chr8|20197542|20204083|+2|r6|T4
ASAIQFLQFPLVNGAAPELPVPTGGPAVGAR q value: 0.009854 Tr_novel:TRUE RefSeq_Novel:TRUE
Search result spec prec mz: 1016.5635 Actual spec prec mz: 1016.5635
Fragments matched per AA: 1.81 Proportion of top 20 peaks matched: 0.2
Intensity
6e+05
4e+05
2e+05
0e+00
y15
y12_
y12
y10
y16
y13
y17
y18
b20*
b25*
b29_
M/Z
500
1000
1500
B
Scatterplot of predicted elution time
Fitting R2: 0.724
Novel peptide residual Z score: −2.58
Number of peptides: 2747
Pred. hydrophobicity
60
40
20
0
0
20000
40000
Experimental scan number
C
Distributions of residuals from best−fit line
of predicted RT vs Expt. scan number
Line: Z score of novel peptide
Z: −2.58
count
800
600
400
200
0
−10
−5
0
5
10
Z scores of residuals from best linear fit
